# Anti-inflammatory effect of naringin and sericin combination on human peripheral blood mononuclear cells (hPBMCs) from patient with psoriasis

**DOI:** 10.1186/s12906-019-2535-3

**Published:** 2019-07-10

**Authors:** Raksawan Deenonpoe, Pokpong Prayong, Nattakarn Thippamom, Jitlada Meephansan, Kesara Na-Bangchang

**Affiliations:** 1 Chulabhorn International College of Medicine, Thammasat University, Rangsit Campus , Pathum Thani, 12120 Thailand; 2 Present address: Department of Pathology, Faculty of Medicine, Khon Kaen University, Khon Kaen, 40002 Thailand; 3 Faculty of Thai Traditional and Alternative Medicine, Ubon Ratchathani Rajabhat University, Ubon Ratchathani, 34000 Thailand; 4 Faculty of veterinary medicine, Western University, Kanchanaburi Campus, Kanchanaburi, 71170 Thailand; 5 Division of Dermatology, Chulabhorn International College of Medicine, Thammasat University, Rangsit Campus , Pathum Thani, 12120 Thailand; 6 Center of Excellence in Pharmacology and Molecular Biology of Malaria and Cholangiocarcinoma, Thammasat University, Rangsit Campus , Pathum Thani, 12120 Thailand

**Keywords:** Psoriasis, Naringin, Sericin, Immunomodulation, Cytokines, Anti-inflammatory

## Abstract

**Background:**

Several immunological pathways, particularly skin inflammation via various pro-inflammatory cytokines have been reported to be involved in the pathogenesis and clinical manifestations of psoriasis. The aim of the study was to investigate the potential role of naringin from *Citrus maxima* (Burm.) Merr and sericin from *Bombyx mori* combination in the treatment of psoriasis. Inhibitory effects on the expression of mRNA and the production of pro-inflammatory cytokines (TNF-α, IL-6, IL-23, and IL-12p40) were investigated.

**Methods:**

Human peripheral blood mononuclear cells (hPBMCs) were isolated from 10 healthy subjects and 10 patients with psoriasis. The hPBMCs from each group were exposed to naringin or sericin alone, and the combination of naringin and sericin. The expression levels of mRNA and the production of all cytokines were determined using quantitative RT-PCR and ELISA, respectively.

**Results:**

Naringin/sericin combination significantly decreased the expression of mRNA and the production of all pro-inflammatory cytokines in hPBMCs from patients with psoriasis. The potency of inhibitory activity was markedly higher than naringin or sericin alone.

**Conclusion:**

The activity of naringin/sericin combination on down-regulation of these pro-inflammatory cytokines suggested its potential clinical use in psoriasis as well as other inflammation-associated diseases. The combination might be used as a complementary therapy with conventional treatment in psoriasis to improve clinical efficacy and tolerability.

## Background

Psoriasis is a chronic, autoimmune skin disorder and multifactorial disease caused by various environmental, genetic, and immunological factors [1]. The prevalence of psoriasis worldwide is 1.3–2.2% [2]. The disease is characterized by epidermal hyperplasia, inflammatory cell infiltration, vascular remodeling and erythematous plaques, plaque psoriasis, pustular psoriasis, and guttate psoriasis [3]. Pathogenesis of psoriasis remains unclear, but the association between psoriasis and other autoimmune diseases has been the subject of ongoing research. Interleukin (IL)-23/Th17 cell axis plays a crucial role in the pathogenesis of psoriasis [4, 5]. In the initiation phase following trauma or infection, keratinocytes release the antimicrobial peptide LL37. This peptide binds to self-DNA and self-RNA fragments that are released by dying or stressed skin cells. The complexes activate plasmacytoid dendritic cells (pDCs) to produce type I interferons (IFNs), which in turn, activate myeloid DCs (mDCs) through Toll-like receptor 8 (TLR8) [6]. IL-23 and IL-12 that are released from the activated mDCs then activate Th17, Th1, and Th22 cells to produce various cytokines such as IL-6, IL-17, IL-22, IFN-γ, and tumor necrosis factor (TNF) [7, 8]. These cytokines promote stimulation of keratinocytes to amplify the inflammation typically observed in psoriatic lesions [9]. Current treatment of psoriasis is limited by adverse drug reactions/toxicity, recurrence, and drug resistance.

Herbal medicines have been used as an alternative treatment for various diseases or pathological conditions, including psoriasis. Several Thai medicinal herbs have been used for the treatment of psoriasis, including *Alpinia galanga*, *Curcuma longa*, and *Annona squamosa* [10]. In addition, naringin extracted from citrus plants is a rich source of flavonoids with various pharmacological benefits such as anti-inflammatory activity and inhibitory activity on chemokine production associated with psoriasis pathogenesis [11]. Moreover, naringin has been proved to exert positive effects on hypertension, hyperlipidemia, hyperglycemia, and obesity [12]. The anti-inflammatory and wound healing activities of 1, 2, and 4% (w/w) naringin ointment have been reported [13]. The mechanism is through triggering the down-regulated expression of NF-κB, TNF-α, IL-1β, IL-6, IL-8, and up-regulated expression of VEGF and TGF-β, which promote tissue repair in an animal model [9]. These support the potential of naringin for further development as an alternative medicine for prevention and treatment of diseases involving abnormal metabolism.

Nevertheless, safety and pharmacokinetics and pharmacodynamics issues should be of concern in the further development of naringin for clinical uses. Sericin, a natural polymer produced from the silkworm *Bombyx mori*, has been shown to exhibit anti-inflammatory and inhibitory activities on IFN-γ, IL-10, and TNF-α production [14]. At present, sericin was widely investigated on large scale industry as the application in biomedical and therapeutical fields of traditional medicine [10]. Naringenin (aglycone of naringin) loaded with sericin has been shown to inhibit TNF-α expression [11]. The aim of the study was to investigate the potential role of naringin/sericin combination for the treatment of psoriasis through inhibition of the expression of mRNA and the production of cytokines involved in the inflammatory process (TNF-α, IL-6, IL-23, and IL-12p40) [11] to use in complementary medicine. Herbal medicines have been used as an alternative treatment for various diseases or pathological conditions, particularly our unusual disease, psoriasis. Many Thai medicinal herbs were used to be anti-psoriatic extract, including *Alpinia galanga*, *Curcuma longa*, *Annona squamosa* [12]. Also, naringin extracted from citrus plants is a rich source of flavonoids with various pharmacological benefits such as anti-inflammatory activity and inhibitory activity on chemokine production related psoriasis development [13].

Moreover, naringin has been proved to have positive effects on hypertension, hyperlipidemia, hyperglycemia, and obesity [14]. The anti-inflammatory and wound healing activities of 1, 2, and 4% (w/w) naringin ointment have been reported [15]. The mechanism is through triggering down-regulated expression of NF-κB, TNF-α, IL-1β, IL-6, IL-8, and up-regulated expression of VEGF and TGF-β, which promote tissue repair in an animal model [9]. These support the potential of naringin for further development as an alternative medicine for prevention and treatment of diseases involving abnormal metabolism. Nevertheless, safety and pharmacokinetics and pharmacodynamics issues should be of concern in the further development of naringin for clinical uses. Sericin, a natural polymer produced from the silk worm *Bombyx mori*, has been shown to exhibit anti-inflammatory and inhibitory activities on IFN-γ, IL-10, and TNF-α production [14]. At present, sericin was widely investigated on large scale industry as the application in biomedical and therapeutical fields of traditional medicine [10]. Naringenin (aglycone of naringin) loaded with sericin has been shown to inhibit TNF-α expression [11]. The aim of the study was to investigate the potential role of naringin/sericin combination for the treatment of psoriasis. Inhibitory effects on the expression of mRNA and the production of pro-inflammatory cytokines TNF-α, IL-6, IL-23, and IL-12p40 [11] was investigated to use in complementary medicine [15].

## Methods

### Preparation of naringin and sericin

One of the subspecies of *Citrus maxima* (Burm.) Merr. including Kao-Yai was purchased from the local market (Talaad Thai Market) in Pathumthani, Thailand during February 2016. All plant samples were taxonomically identified and authenticated by Dr. Pokpong Prayong (Faculty of Thai Traditional and Alternative Medicine, Ubon Ratchathani Rajabhat University), and the voucher specimens were deposited in the Herbarium of the Faculty of Pharmaceutical Sciences, Khon Kean University, Thailand. The extraction procedure was according to Sudto et al. [16]. The albedo (spongy white interior) and flavedo (green exterior) parts of the peel were separated and cut into small pieces, and sun-dried for 3 days. The samples were placed in an oven (40–50 °C, 24 h) and ground into powder. The dry powder was macerated in methanol for 3 days. The slurry was filtered, and the extract was dried under reduced pressure using a rotary evaporator. Water was added to the dry extract and stirred at 70 °C for 30 min and transferred into a separating funnel. Dichloromethane was added, and the mixture was swirled and left over at 25 °C for 3–4 days to allow crystallization of naringin in the aqueous layer. The naringin crystals were collected by filtration through filter paper and air-dried. The identity of the naringin crystals was determined by the reflective index under infrared (IR) spectroscopy. Infrared spectroscopy was used for chemical characterization of the investigated herbs. The spectra of naringin (15 mg) were obtained using the IR Shimaszu® model 8001 spectrophotometry in the region of 4000–600 /cm spectrum.

Sericin was purchased from Ruenmai-Baimon Co. Ltd. Surin Province, Thailand. The dried powder was analyzed using the previously described method [17]. Sericin was separated from the cocoons of *Bombyx mori* by boiling with water at 110–120 °C for 30–40 min. The samples were placed in an oven-dried (40–50 °C, 24 h) and ground into power. Analysis of the amino acid profile revealed the purity of sericin dried power of 100%.

### Sample collection

Blood sample collection was performed at Thammasat Chalermprakiat Hospital, Pathumthanee during January–August 2018. The study protocol was approved by the Ethics Committee of Thammasat University (MTU-EC-OO-2-047/60). Heparinized blood samples (5 mL each) were collected from (i) 10 healthy subjects (5 males and 5 non-pregnant females, aged 20–60 years) with no history of previous treatment with anti-inflammatory drugs within the past 2 months; and (ii) 10 patients (5 males and 5 females, aged 20–60 years) with mild degree psoriasis (Psoriasis Area Severity Index 2–8) with no history of previous treatment with anti-inflammatory drugs, phytotherapy, or any treatments of psoriasis within the past 2 months.

### Isolation of human peripheral blood mononuclear cells (hPBMCs)

hPBMCs were separated from blood samples within 6 h after collection using Ficoll-Paque™ (GE Healthcare, NJ, USA). In brief, the blood sample was diluted with 2 x volume of 1x PBS (pH 7.4) and carefully layered over an equal volume of Ficoll-paque in a 15 mL-conical tube. The suspension was centrifuged (400 xg, 30 min, 20 °C) and the upper layer was aspirated, leaving the mononuclear cell layer (lymphocytes, monocytes, and thrombocytes) undisturbed at the interphase. The mononuclear cell layer was transferred to a new conical tube, and 1x PBS was added. Following centrifugation (300 xg, 10 min, 20 °C), the supernatant was carefully removed. The cell pellets were resuspended with 1x PBS. Following centrifugation (200 xg, 10 min, 20 °C), the supernatant was carefully removed. The hPBMC pellets were obtained through centrifugation over Ficoll–Paque™ cushions of buffy-coat, and cell number was counted using a cell counter. The cell suspension at density of 5 × 10^6^ cells/mL was prepared in RPMI 1640 medium supplemented with 10% fetal bovine serum (RPMI-FBS) and incubated at 37 °C for 3 h.

### Optimization of naringin and sericin concentrations

The hPBMCs from each healthy subjects and psoriasis patients (100 μL of 1 × 10^5^ cells/mL/well of a 24-wells microtiter plate in RPMI-FBS and glucose) was exposed (37 °C, 24 h) to each compound (160, 80, 40, 20 μg/mL and 800, 400, 200, 100 μg/mL for naringin and sericin, respectively). These concentration ranges used were based on the previously reported IC_50_ values of narigin (8.7–33.6 μg/mL) and sericin (> 200 μg/mL) [10]. The concentrations of both compounds that did not produce significant cytotoxicity (< 50% of control) were determined. After centrifugation (10,620 xg, 5 min, 4 °C), the pellets were kept in a mixture of 90% FBS and 10% DMSO. Both the pellets and supernatant were stored at − 80 °C until use. The cytotoxic effects of naringin, sericin, and naringin/sericin combination were investigated using MTT (3-(4,5-dimethylthazolk-2-yl)-2,5-diphenyl tetrazolium bromide) cell proliferation assay [15]. The assay measures the cell proliferation rate and conversely when metabolic events lead to apoptosis or necrosis, the reduction in cell viability. The hPBMC pellets were exposed to various concentrations of naringin (160, 80, 40, and 20 μg/mL), or sericin (800, 400, 200, and 100 μg/mL) for additional 23 h (37 °C). Control cells were treated with RPMI 1640 and 10% FBS. The blue MTT dye was added, and the fluorescence of the medium of each well was measured (excitation 530–560 nm, and emission 590 nm) after 4 h of exposure. Cytotoxic effect of each compound was expressed as percent inhibition of cell viability compared with control:

Inhibition of cell viability (%) = 100 - (fluorescence of treated hPBMC/ fluorescence of control hPBMC) × 100.

The concentrations of naringin and sericin that did not produce significant cytotoxicity (< 50% of control) were found to be 20 and 100 μg/mL, respectively.

### Exposure of hPBMCs to naringin and sericin extracts and cell proliferation assay

The hPBMCs (1 × 10^5^ cells/mL/well in RPMI-FBS) from samples from healthy subjects (*n* = 10) and psoriasis patients (*n* = 10) were incubated with naringin (20 μg/mL), sericin (100 μg/mL), or naringin (20 μg/mL)/sericin (100 μg/mL) combination (1:1, v:v) for 24 h (37 °C). The control cells (untreated control) for each group were treated with RPMI-FBS. Following centrifugation (10,620 xg, 5 min, 4 °C), the pellets were kept in a mixture of 90% FBS and 10% DMSO. Both the pellets and supernatant were stored at − 80 °C until use.

Cell proliferation assay was performed to confirm the concentrations of naringin and sericin that resulted in < 50% cell death using CFSE Cell Division Tracker Kit (BioLegend®, San Diego, USA) [18]. The cells (10-100 × 10^6^ cells/mL in 5 μM CFSE working solution) were incubated at room temperature (25 ^o^ C) for 20 min (protected from light). The staining was quenched by adding five times the original attaining volume of cell culture medium containing 10% FBS. The PBMC pellets were resuspended in prewarmed cell culture media and incubated at 37 ^o^ C for 10 min. The CFSE-labeled hPBMCs were analyzed by flow cytometry (BD FACSVERSE®). Data analysis was performed using BD FACSUite software. CFSE-unlabeled hPBMCs were used as background at 10^2^ cells, and the positive staining with living cells was determined as a percent of total cell proliferation.

### Determination of mRNA expression levels of pro-inflammatory cytokines

For determination of mRNA expression levels of TNF-α, IL-6, IL-12p40, and IL-23 genes, total RNA was isolated from hPBMCs using GF-1 Total RNA Extraction Kit (Vivatis^R^, Selangor Darul Ehsan, Malaysia). Total RNA concentration of each gene was measured spectroscopically (260 nm) and adjusted to the final concentration of 1 μg/μL. The RNA was used in a two-step real-time PCR kit (Vivantis^R^) to provide a reliable synthesis of full-length cDNA. The cDNA was amplified by real-time PCR using KAPA SYBR® FAST qPCR Master Mix (2x) Kit (Kapa Biosystems, Cape Town, South Africa). Each desired cDNA fragment was amplified for 40 cycles using each gene-specific primer pair as listed in Table 1.

**Table 1:** Specific primers used for determination of mRNA expression of the pro-inflammatory genes

Gene	Sequences	References
IL-12p40	F-5′-TGGAGTGCCAGGAGGACAGT-3′ R-5′-TCTTGGGTGGGTCAGGTTTG-3’	Ji Hoon Chun et al. (2017) [19]
IL-23	F-5′-GTATCCAGTGTGAAGATGGTTGTGA-3′ R-5′-CGGA TCCTTTGCAAGCAGAA-3’	Hsin-Hua Chen et al. (2016) [20]
TNF-α	F-5′-GCTGCTCACCTCATTGGAG-3′ R-5′-CCAGGAGAGAATTGTTGCTCA-3′	Aleksandra Batycka-Baran (2016) [21]
IL-6	F-5’- CCTCTCTGCAAGAGACTTCCAT-3′ R-5’- AGTCTCCTCTCCGGACTTGT-3’	Jun Sun et al. (2013) [22]
GAPDH	F-5’-GGGCTCTCTGCTCCTCCCTGT-3′ R-5′-CGGCCAAATCCGTTCACACCG-3′	Jun Sun et al. (2013) [22]

The CT values of naringin and sericin treated hPBMCs were calculated, and data were expressed as the fold change of untreated control (healthy) using 2^-(∆∆ Ct)^.

### Determination of pro-inflammatory cytokine production

The levels of pro-inflammatory cytokines TNF-α, IL-6, IL-12p40, and IL-23 were measured in hPBMCs supernatant isolated from healthy subjects and patients with psoriasis following exposure to naringin, sericin, or naringin/sericin combination. The conditioned medium was removed from a 24-well plate after 24 h incubation and stored at − 80 °C until use. The concentration of each cytokine was determined according to the manufacturer’s instructions (BioLegend®, Advanced Medical Science, San Diego, CA). In Brief, the supernatant in each well was washed four times with 1x wash buffer. Diluted standard solution of each cytokine (100 μL each) was added to the reference standard wells, whereas hPBMC supernatant (100 μL) was added to the sample wells. Following incubation for 3 h (with agitation), each well was washed four times with 1x wash buffer and antibody solution (100 μL) was added. The plate was incubated (with agitation) at room temperature (25 °C) for an additional 1 h. The content of each well was washed four times, and Avidin-HRP solution (100 μL) was added. The plate was incubated (with agitation) at room temperature for an additional 30 min. The well content was washed five times with 1x wash buffer and substrate solution F (100 μL), and the plate was incubated at 25 °C for 30 min in the dark. The reaction was stopped by adding stop solution (100 μL) to each well and absorbance measured spectroscopically (450 nm and 570 nm). The concentrations of each cytokine in the samples were determined from the calibration curve.

### Statistical analysis

Statistical analysis was performed using SPSS software (version 19, IBM, USA). Quantitative data were expressed as median and range of samples from each group (*n* = 10 each) in three independent experiments (triplicate each). Difference between groups was determined using the Mann-Whitney U test at a statistical significance levels (α) of 0 .05 and 0.001.

## Results

### Preparation of naringin and sericin

**Fig. 1:**
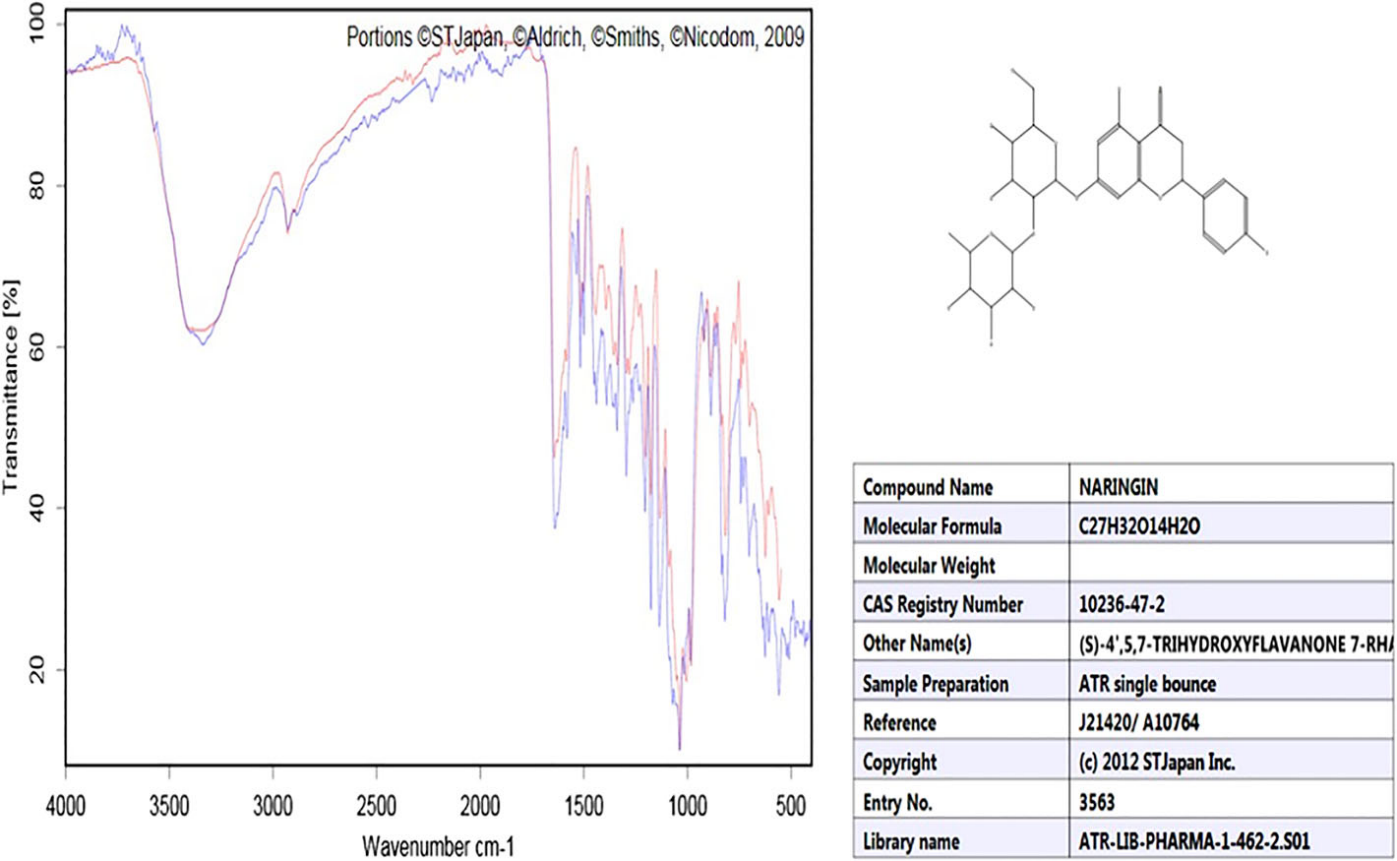
The reflective index (RI) of naringin measured by infrared (IR) spectroscopy confirmed naringin as the main compound from citrus peel extract

Spectrophotometry in IR region is based on the principle of interaction of electromagnet- tic radiation that passes through the sample and is absorbed by the links, leading to stretching or folding of these links [23]. The absorption spectra obtained from naringin in the IR region are shown in Fig. 1. The characteristic spectra of naringin (% yields = 2.1% w/w) used in the study were divided into two regions, i.e., 4000–1300 cm^− 1^ (the region of functional groups), and 1300–600 cm^− 1^ (the region of the molecule fingerprint) [24, 25]. The attributions of the characteristic bands of the molecule were established based on the literature (Table 2). Spectroscopy in the IR region confirmed the naringin flavonoid structure based on the presence of all characteristic bands.

**Table 2:** Attributions of the main bands of the naringin spectrum

Attribution	Frequency (cm^− 1^)
OH (axial deformation)	3398.53
	1036.79
C=O	1626.21
C=C	1588.47
Aromatics	~ 1200
Axial deformation of C-O-C	985.19
Angular deformation C-H	~ 800

Sericin powder provided by the manufacturer had previously been confirmed to be non-toxic. Analysis of the amino acid profile of the sericin powder revealed serine (33,429.89 mg/100 g) and aspartic acid (18,487.66 mg/100 g) as prominent amino acids.

### Optimization of naringin and sericin concentrations

The inhibitory effect of naringin on hPBMCs viability was higher than 50% at the concentration range 40–160 μg/mL. At 20 μg/mL however, the inhibitory effect was lower than 50%. For sericin, the inhibitory effect on hPBMCs viability was higher than 50% at the concentration range 200–800 μg/mL, but lower than 50% at 100 μg/mL. The mixture of naringin (20 μg/mL) and sericin (100 μg/mL) at the ratio of 1:1 (v:v) produced an inhibitory effect on hPBMCs viability of lower than 50%. Inhibitory effects on the viability of hPBMCs from healthy subjects following exposure to naringin (160 μg/mL)/sericin (800 μg/mL), naringin (80 μg/mL)/sericin (400 μg/mL), naringin (40 μg/mL)/sericin (200 μg/mL), and naringin (20 μg/mL)/sericin (100 μg/mL) combination were 77.78, 65.4, 52.3 and 21.52%, respectively. The corresponding inhibitory effects on the viability of hPBMCs from psoriasis patients were 79.5, 68.9, 55.6 and 22.40%, respectively.

### Exposure of hPBMCs to naringin and sericin and cell proliferation assay

**Fig. 2:**
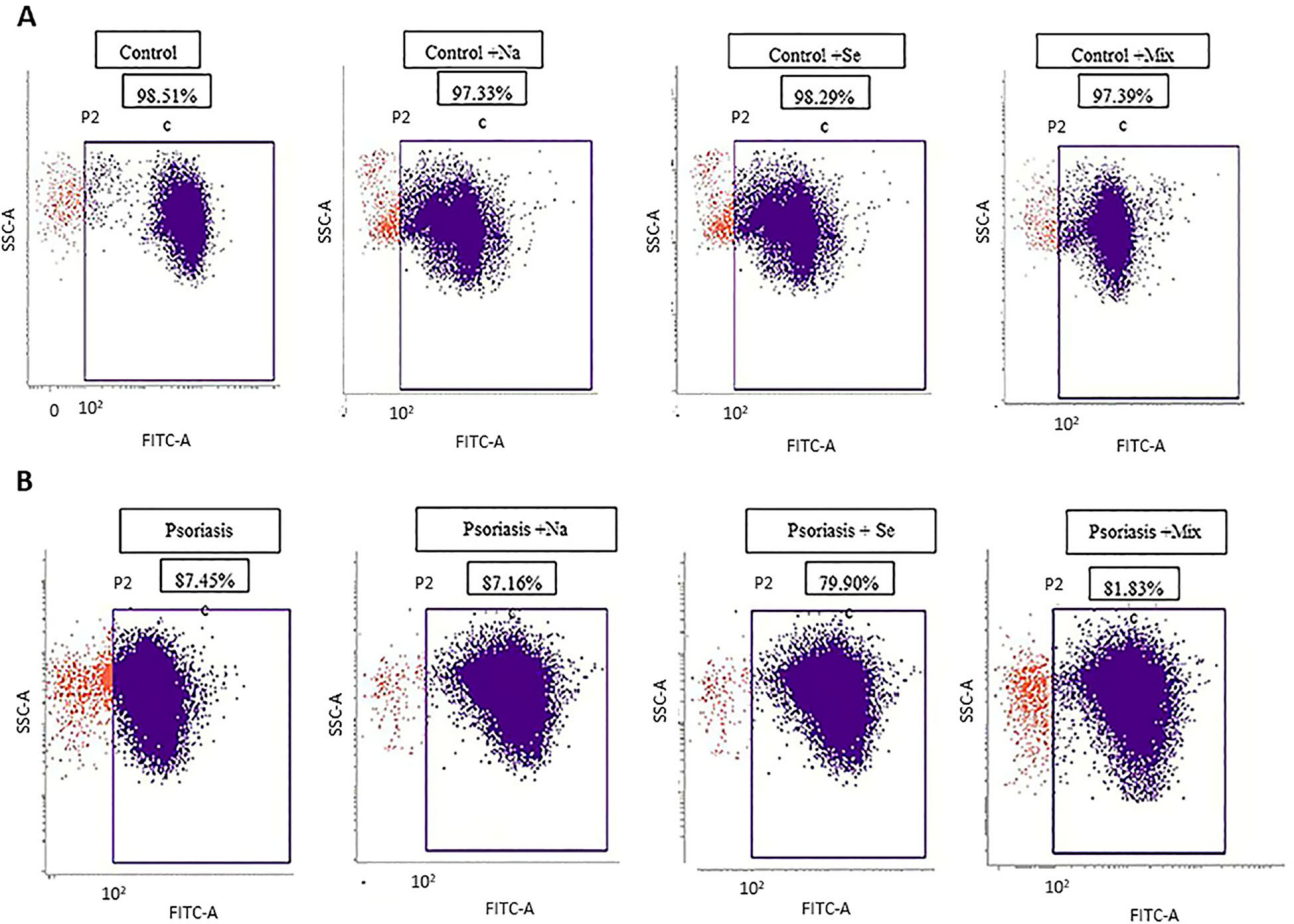
The viability of the hPBMC (measured by flow cytometry) isolated from blood of healthy subjects (control) (**a**) and psoriasis patients (**b**) following exposure to 20 μg/ml Naringin (Na), 100 μg/ml Sericin (Se), and Naringin/Sericin mixture 20/100 μg/ml compare with control (hPBMC from healthy subjects or psoriasis patients without exposure)

In the control group (samples from healthy subjects), the hPBMCs viability as confirmed by flow cytometry was not significantly suppressed by naringin (20 μg/mL), sericin (100 μg/mL), or naringin (20 μg/mL)/sericin (100 μg/mL) combination compared with untreated samples (97.33, 98.29, 97.39% vs. 98.51%, respectively, *P* > 0.05). Likewise, in the psoriasis group, the hPBMCs viability was not significantly suppressed by naringin, sericin, or naringin/sericin combination at the same concentrations compared with untreated samples (87.16, 79.90, 81.83% vs. 87.45%, respectively, *P* > 0.05) as shown in Fig. 2

### Determination of mRNA expression levels of pro-inflammatory cytokines

**Fig. 3:**
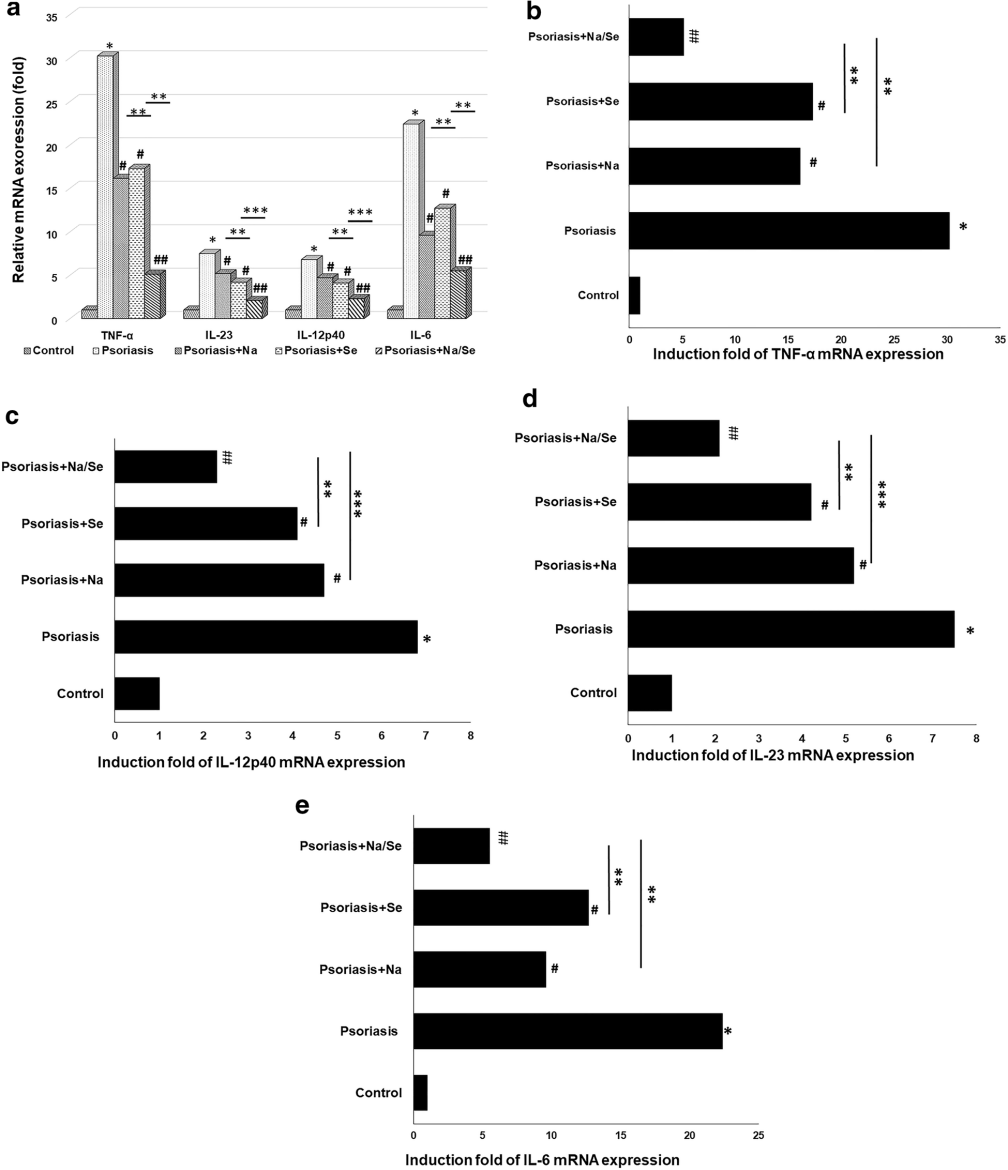
The mRNA expressions levels of TNF-α, IL-6, IL-12p40 and IL-23 genes in hPBMCs obtained from psoriasis patients and healthy control treated with 20 μg/mL Na, 100 μg/mL Se and Na/Se combination (20/100 μg/mL at the ratio of 1:1 (v:v). The data showed the effect of naringin (Na) and sericin (Se) on hPBMCs-induced pro-inflammatory cytokine mRNA expression in hPBMCs (**a**). The patterns of induction fold of mRNA expression of all cytokines generally correlated with mRNA expression levels (**b**-**e**). *P < 0.001 vs. *untreated control hPBMCs from healthy subjects by Mann-Whitney U test. #P < 0.05 and ## P < 0.001* vs. *untreated pPBMCs from psoriasis patients by Mann-Whitney U test*. ** P < 0.05 and *** P < 0.001 vs. *treated hPBMC with Na/Se from psoriasis patients by Mann-Whitney U test*

The mRNA expression levels of TNF-α, IL-6, IL-12p40 and IL-23 genes in hPBMCs obtained from psoriasis patients were significantly higher than that from healthy control (30.22, 7.5, 6.8 and 22.4 fold, respectively, *P* < 0.001). The mRNA expression levels of all cytokines were significantly lower in hPBMCs from psoriasis patients following exposure to naringin (20 μg/mL) or sericin (100 μg/mL) alone compared with untreated control (psoriasis patients) (*P* < 0.05). The effect was more prominent following exposure to naringin/sericin combination (*P* < 0.001) (Fig. 3a). The patterns of induction fold of mRNA expression of all cytokines generally correlated with mRNA expression levels (Fig. 3b-e). The induction fold of TNF-α following exposure to naringin or sericin alone was significantly lower than the untreated hPBMCs from psoriasis patients (16.14, 17.3 and 30.22 fold, respectively) and the effect was more prominent with naringin/sericin combination (5.12 fold) (Fig. 3b). The induction fold of IL-12p40 following exposure to naringin or sericin alone was also significantly lower than the untreated hPBMCs from psoriasis patients (4.7, 4.1, and 6.8 fold, respectively) and the effect was markedly seen with naringin/sericin combination (2.09 fold) (Fig. 3c). The induction fold of IL-23 following exposure to naringin or sericin alone was significantly lower than the untreated hPBMCs from psoriasis patients (5.18, 4.2 and 7.5 fold, respectively) and the effect was markedly seen with naringin/sericin combination (2.3 fold) (Fig. 3d). The induction fold of IL-6 following exposure to naringin or sericin alone was significantly lower than the untreated hPBMCs from psoriasis patients (9.6, 12.7 and 22.4 fold, respectively) and the effect was markedly seen with naringin/sericin combination (5.5 fold) (Fig. 3e).

### Determination of pro-inflammatory cytokine production

**Fig. 4:**
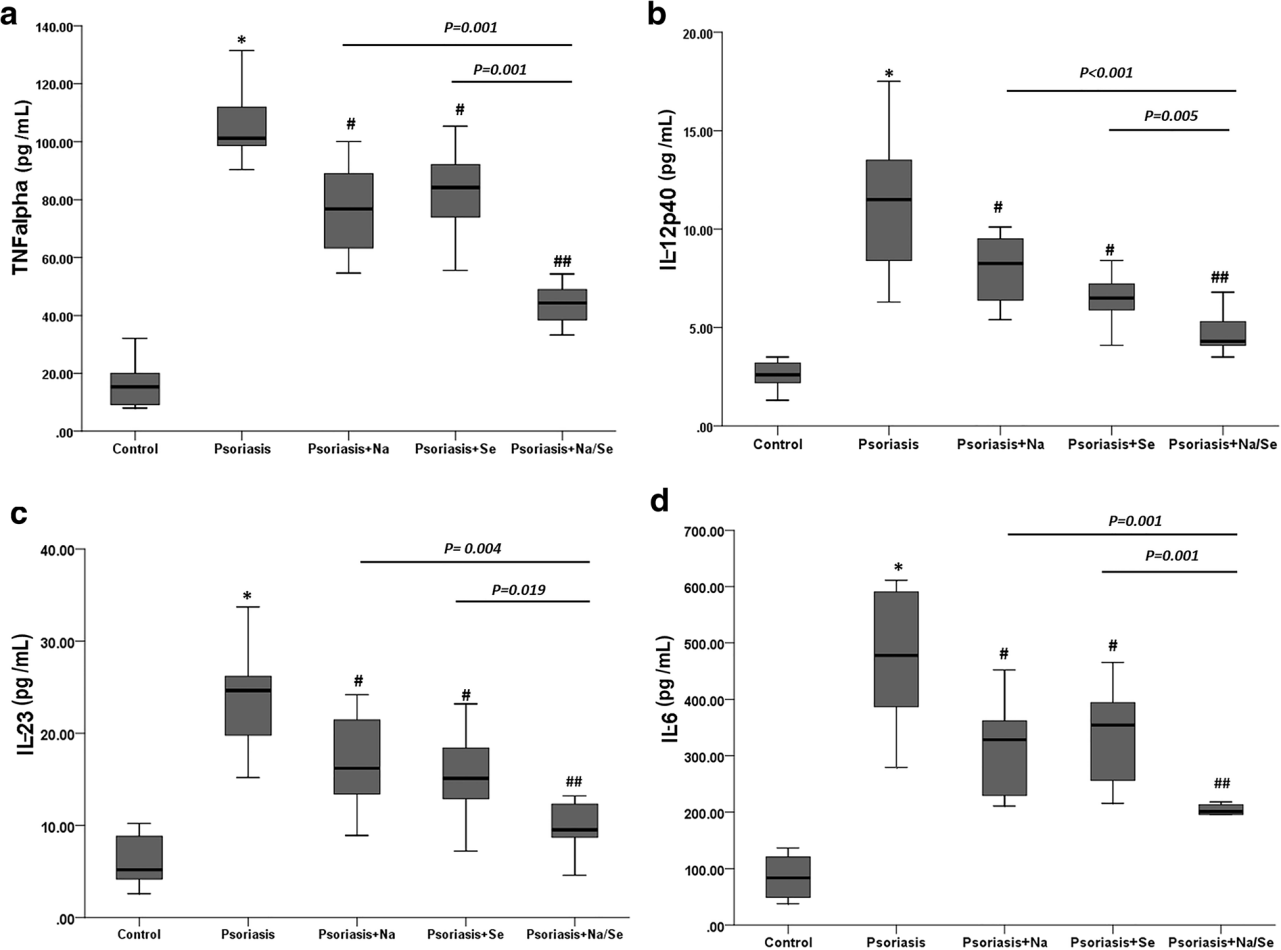
Effect of naringin (Na) and sericin (Se) on the induction of the production of (**a**) TNF-α, (**b**) IL-12p40, (**c**) IL-23, and (**d**) IL-6. The hPBMCs from healthy subjects (untreated control) and patients with psoriasis were treated with 20 μg/mL Na, 100 μg/mL Se and Na/Se combination (20/100 μg/mL at the ratio of 1:1 (v:v). The cytokine production was measured by ELISA. Data are expressed as median (range) of 10 samples in each group (3 independent experiments, triplicate each). *P < 0.001 vs. *untreated control hPBMCs from healthy subjects by Mann-Whitney U test. #P < 0.05 and ## P < 0.001* vs. *untreated pPBMCs from psoriasis patients by Mann-Whitney U test. One* vs. *untreated pPBMCs from psoriasis patients by Mann-Whitney U test*

The production of pro-inflammatory cytokines TNF-α, IL-6, IL-12p40, and IL-23 of the hPBMCs from the psoriasis patients were significantly higher than that of the healthy subjects (*P* < 0.001). The levels of all cytokines were significantly lower in hPBMCs following exposure to naringin (20 μg/mL), or sericin (100 μg/mL) alone compared with healthy control. This effect was even markedly observed in hPBMCs treated with naringin/sericin combination (*P* < 0.001). The patterns of the production of all cytokines were generally similar. The inhibitory effect of naringin on TNF-α, IL-12p40, IL-23, and IL-6 production appeared to be also similar to sericin. The production of TNF-α in hPBMCs following exposure to naringin/sericin combination was significantly lower than naringin or sericin alone [median (range): 44.8 (33.3–74.5), 76.8 (54.6–100.1) and 84.2 (55.5–105.4) pg/mL*,* respectively] (Fig. 4a). The level of IL-12p40 in hPBMCs following exposure to naringin/sericin combination was also significantly lower than naringin or sericin alone [median (range): 4.3 (3.5–6.8), 8.25 (5.4–10.1) and 6.5 (5.5–9.2) pg/mL*,* respectively*,* respectively] (Fig. 4b). The production of IL-23 following exposure to naringin/sericin combination was significantly lower than naringin or sericin alone [median (range): 9.5 (4.6–20.2), 16.2 (8.9–24.2) and 15.1 (7.2–23.2) pg/mL, respectively] (Fig. 4c). Similarly, the production of IL-6 in hPBMCs following exposure to naringin/sericin combination was also significantly lower than naringin or sericin alone [median (range): 200.9 (109.6–303), 328.5 (210.6–425.6) and 354.6 (215.5–465) pg/mL*,* respectively] (Fig. 4d)

## Discussion

Several immunological pathways and antigen-presenting cell (APC) and T-cell have been reported to be involved in the pathogenesis and clinical manifestations of psoriasis [26]. Interestingly, the results of the present study revealed a significant increase in the expression and production of TNF-α level from the hPBMCs from patients with psoriasis compared with healthy subjects. The major role of tumor necrosis factor (TNF-α) is regulation of antigen-presenting cells [27]. This cytokine is not only prominent in normal skin, but also damaged keratinocytes [26]. Apart from TNF-α, interleukin-6 (IL-6), interleukin-12 (IL-12) and interleukin-23 (IL-23) are also involved in the pathogenesis and clinical manifestations of psoriasis. IL-23 appears to be the important driver behind the T17, which is an intermediate molecule to stimulate keratinocyte proliferation [22, 28, 29]. Interleukin-12 (IL-12) is a heterodimer of IL-23 that stimulates Th1 cells, and its level is usually elevated in patients with psoriasis [5, 30]. IL-6 is a pro-inflammatory cytokine produced by several types of immune and non-immune cells [31]. The keratinocyte also produces IL-6, which is involved in proliferative response [26]. In the present study, IL-6, IL-12p40, and IL-23 mRNA were shown to be over-expressed, and the levels of these cytokines were also increased in the psoriasis group. The production of IL-6 was found to be highest among these three interleukin cytokines. The level of IL-6 was shown to be increased in several physiological and pathological conditions such as circadian secretion associated with sleep and stress [31]. Any factor that raises the level of IL-6 could be a risk factor in stimulating the pathogenesis of psoriasis. All of these cytokines work as a network that leads to pathogenesis and clinical manifestation of psoriasis [19, 32]. The mechanism is through the activation of T-cell and NF-kB [20]. Discovery of new immunological factors involved in pathogenesis and clinical manifestations of psoriasis is of interest as new drug targets [21, 33].

Some Thai herbs, including naringin isolated from citrus peel [34] have been shown to exhibit anti-psoriatic activity with potent anti-inflammatory and antioxidant activities [11, 35]. Moreover, the silk protein sericin has also been shown to exhibit anti-inflammatory activity through inhibition of pro-inflammatory cytokines involved in the pathogenesis of psoriasis [15].

The ability of naringin/sericin combination to suppress the production of pro-inflammatory cytokines TNF-α, IL- 6, IL-12p40 and IL-23 is clinically benefit. The reduction of these cytokines might continually decrease, facilitating further T cell/keratinocyte interactions. Therefore, this immunosuppression would result in inhibition of epidermal hyperproliferation with elongation of the rete ridges, parakeratosis, and chemotactic for neutrophil that play important role in the pathogenesis of psoriasis.

Our present study focused on the anti-inflammatory activity of naringin and sericin when used in combination. Both naringin and sericin alone significantly decreased the expression of mRNA and the production of TNF-α, IL-6, IL-23, and IL-12p40 by hPBMCs from psoriasis patients, with similar potency of activity. Results suggest that the anti-inflammatory activity observed following exposure of hPBMCs to naringin and sericin (24 h) could be via suppression of mRNA expression and production of TNF-α, IL-6, IL-12p40 and IL-23 cytokines in psoriasis patients. Nevertheless, naringin appeared to exhibit a preferential inhibitory effect on TNF-α and IL-6 production, while sericin exhibit a preferential inhibitory effect on IL-12p40 and IL-23 production. The mRNA expression levels and the production of the involved cytokines TNF-α, IL-6, IL-12p40, and IL-23 by the hPBMCs following exposure to naringin/sericin combination were investigated in comparison with each compound alone. The combination of naringin and sericin in equal ratio markedly decreased the expression of mRNA and the production of all cytokines by the hPBMCs from psoriasis patients compared with healthy subjects. The potency of activity was significantly higher than naringin or sericin alone. These inhibitory effects were not due to non-specific cytotoxicity on hPBMCs. The high potency of activity of the combination to inhibit mRNA expression and cytokine production would result in improvement of anti-inflammatory activity against psoriasis [36].

Additionally, currently used conventional drugs for psoriasis are relatively expensive. Therefore, the use of herbal medicines would reduce treatment cost. It is noted however, that pharmaceutical formulation of naringin/sericin combination is an issue of concern since both exhibit different solubility characteristics in various solvents [15, 34].

## Conclusions

Results of the study confirmed the superior activity of naringin/sericin combination compared with each compound alone on down-regulation of the pro-inflammatory cytokines including IL-6, IL-12p40, IL-23 and TNF-α related to early inflammation mechanisms of psoriasis pathogenesis. Most importantly, the study confirmed the optimal concentration of the combination that produced anti-inflammatory activity with low acceptable cytotoxic activity. Taken together, results suggest potential clinical use of naringin/sericin combination in psoriasis as well as other inflammation-associated diseases. Development of suitable pharmaceutical formulation of naringin/sericin combination as anti-inflammatory agents for the treatment of psoriasis is required. Efficacy and toxicity, including pharmacokinetics of the combination in the animal model should also be confirmed before clinical trials.

## Data Availability

All data sets used and analyzed during the study are not publicly available. Due to personal or identifying data of patients but are available from the corresponding author on reasonable request.

## Abbreviations

hPBMCs: human peripheral blood mononuclear cells
IL-12p40: Interleukin-12p40
IL-23: Interleukin- 23
IL-6: Interleukin- 6
Na: Naringin
Se: Sericin
TNF-α: Tumor necrosis factor alpha
