# Correction: Anti-inflammatory effect of naringin and sericin combination on human peripheral blood mononuclear cells (hPBMCs) from patient with psoriasis

**DOI:** 10.1186/s12906-023-03945-6

**Published:** 2023-04-12

**Authors:** Raksawan Deenonpoe, Pokpong Prayong, Nattakarn Thippamom, Jitlada Meephansan, Kesara Na-Bangchang

**Affiliations:** 1 Chulabhorn International College of Medicine, Thammasat University, Rangsit Campus, 12120 Pathum Thani, Thailand; 2 Department of Pathology, Faculty of Medicine, Khon Kaen University, Khon Kaen, 40002 Thailand; 3 Faculty of Thai Traditional and Alternative Medicine, Ubon Ratchathani Rajabhat University, Ubon Ratchathani, 34000 Thailand; 4 Faculty of veterinary medicine, Western University, Kanchanaburi Campus, Kanchanaburi, 71170 Thailand; 5 Division of Dermatology, Chulabhorn International College of Medicine, Thammasat University, Rangsit Campus, 12120 Pathum Thani, Thailand; 6 Center of Excellence in Pharmacology and Molecular Biology of Malaria and Cholangiocarcinoma, Thammasat University, Rangsit Campus, 12120 Pathum Thani, Thailand

**Correction: BMC Complement Med Ther 19, 168 (2019)**

10.1186/s12906-019-2535-3

Following publication of the original article [1], the authors reported an error in Fig. 2. The correct figure is given below.

**Fig. 2:**
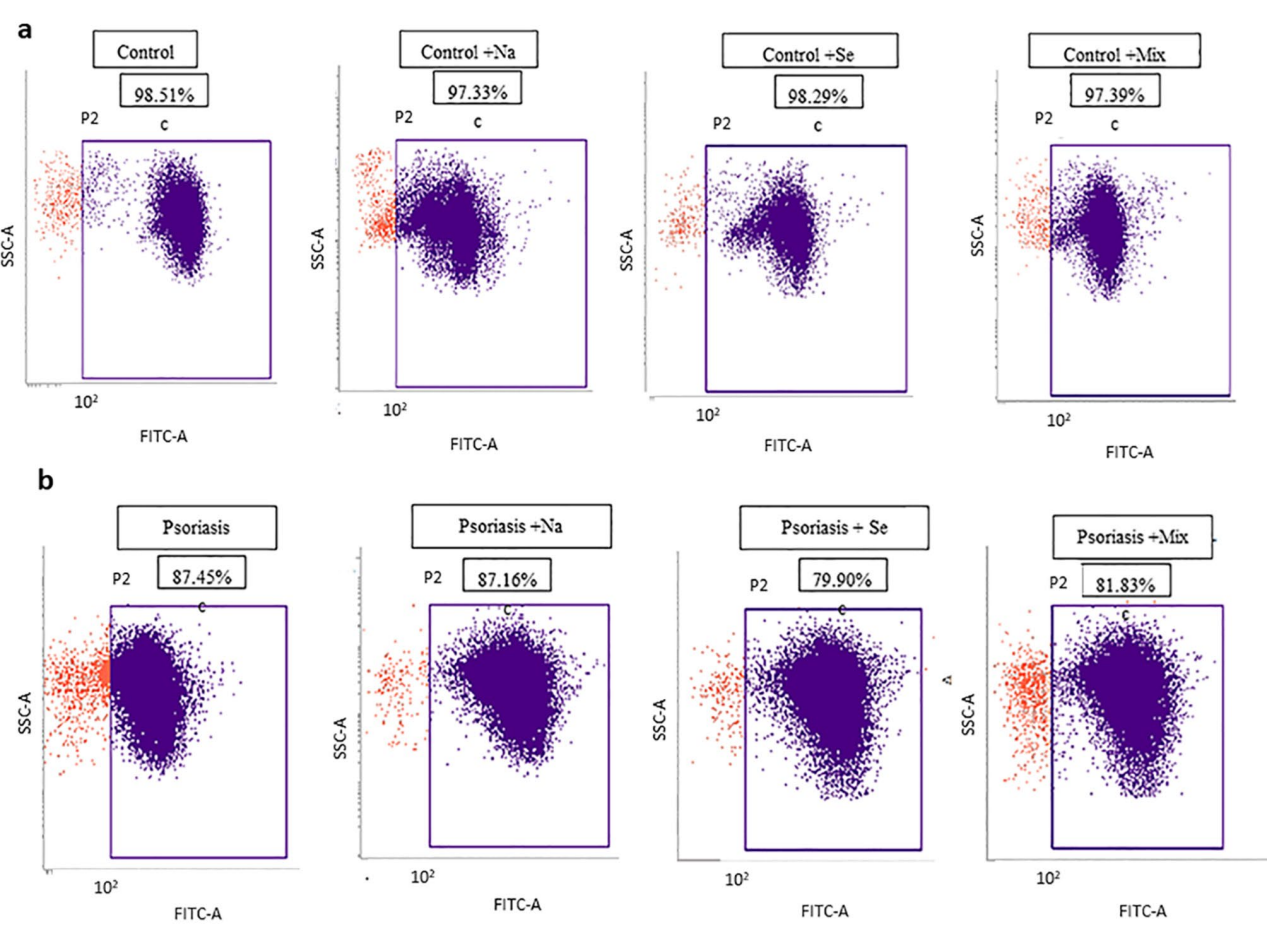
The viability of the hPBMC (measured by flow cytometry) isolated from blood of healthy subjects (control) (**a**) and psoriasis patients (**b**) following exposure to 20 µg/ml Naringin (Na), 100 µg/ml Sericin (Se), and Naringin/Sericin mixture 20/100 µg/ml compare with control (hPBMC from healthy subjects or psoriasis patients without exposure)

The original article [1] has been updated.
